# Medical students’ attitudes towards careers in primary care in Singapore

**DOI:** 10.1186/s12909-020-02377-x

**Published:** 2020-11-25

**Authors:** Humairah Zainal, Helen Elizabeth Smith

**Affiliations:** Department of Family Medicine and Primary Care, Lee Kong Chian School of Medicine, Nanyang Technological University, Singapore, Singapore

**Keywords:** Primary care, General practice, Family medicine, Medical students, Medical school, Singapore

## Abstract

**Background:**

Singapore needs more family doctors to care for its ageing population and their chronic conditions. While there is a shifting of care from acute care settings to more community care, this has not been reflected in the primary care training in local medical schools. Furthermore, no research has explored how different aspects of the medical school curricula in Singapore influence students’ perceptions of careers in General Practice and Family Medicine- a gap that is filled by this study.

**Methods:**

Six focus groups involving 54 students from all three medical schools in Singapore were conducted. Discussions focused on their primary care experience, their professional and career aspirations, and perceptions towards the opportunities and challenges of primary care careers. Qualitative content analysis was used to interpret the data.

**Results:**

The respondents shared eight key concerns of pursuing primary care careers including limited professional opportunities, emphasis on lifestyle benefits rather than professional characteristics, need for business acumen, conflicts created by business in clinical care, mundane case mix, lack of continuity of care, limited consultation time, and specialists’ negative attitudes towards family doctors. The positive views articulated included the opportunities for entrepreneurialism and a portfolio career, breadth of clinical problems presented, and an improved future for primary care.

**Conclusions:**

Improving students’ perceptions of careers in primary care in Singapore would benefit from a concerted effort from multiple stakeholders; medical schools, healthcare providers, professional and regulatory bodies, and government.

## Background

With the increase in the number of complex chronic diseases and in fragmentation of care as a result of specialisation in medicine, more community-based generalists are needed to meet the increasing demand for holistic care for patients [1, 2]. Singapore, with its rapidly ageing population, needs more general practitioners and family physicians (henceforth, family doctors) to care for the elderly and their multi-morbidity [3].^1^ However, in the last decade, while the number of specialist doctors has risen by almost 80%, from 3374 in 2010 to 6010 to date, the number of registered family physicians remains relatively low at 2161 at the point of writing [5, 6].^2^ The number of doctors enrolled onto the Family Medicine Residency Programme has been less than 100 since the programme was introduced in 2011, lower than the number needed each year to meet the Family Medicine needs of the projected population of 6.9 million in 2030 [2].^3^

In 2000, the Ministry of Health (MOH) launched programmes to encourage collaboration between the hospitals and primary care providers. Despite the shift in focus from hospitals towards community care and preventive care, this refocusing of care has not been reflected fully in the delivery of training in local medical schools, and students from all three medical schools in Singapore have limited exposure to primary care settings in their training (Table 1).

**Table 1:** Amount of students’ exposure to primary care settings^a^

School	Preclinical Years^**b**^ (First and second year)	Clinical Years^**c**^ (Third, fourth and fifth year)
National University of Singapore’s Yong Loo Lin School of Medicine (YLL) (Five-year undergraduate programme)	First year: 3 h of attachment in public primary healthcare clinics for each student	Third year: 3 weeks of attachment in public primary healthcare clinics, 2 weeks of GP clinics, 2 days of community hospital for each student Elective programme in fourth year: 2 weeks of public primary healthcare clinics/ GP clinics limited to 30 students
Duke-NUS Medical School (Duke-NUS) (Four-year graduate programme)	Second year: 10 sessions of placement in a GP clinic and 10 sessions in a public primary healthcare clinic	Fourth year: 2 weeks in public primary healthcare clinics and 2 weeks in a community hospital where family physicians practise
Nanyang Technological University’s Lee Kong Chian School of Medicine (LKCMedicine) (Five-year undergraduate programme)	First year: 1 week in a public primary healthcare clinic	Fourth year: 7 weeks in public primary healthcare clinics and private clinics Fifth year: 3-week rotation as student interns in public primary healthcare clinics

^a^Generally, the syllabus focuses on the principles of Family Medicine and clinical approaches to common problems, as well as the practical aspects of consultation, such as basic principles of communication and counselling [2]

^b^The predominance of teaching is theoretical with some practical exposure

^c^Students undergo rotations at hospitals and clinics affiliated with their schools

Simply increasing the number of trained doctors will not automatically expand the General Practice (GP) and Family Medicine (FM) workforce: recruitment initiatives need to be informed by a better understanding of the career aspirations of medical students, their perceptions of the opportunities and challenges of a career in primary care, and how the medical school curricula influence some of these perceptions. Although there are works on how medical schools shape students’ attitudes towards a career in primary care [8–11], they do not discuss in detail the different components of the curriculum; formal, informal and hidden curricula. Additionally, the roles of medical schools, professional bodies and the state tend to be discussed either fleetingly or independently of one another.

Thus, this article explores the ways in which the formal, informal and ‘hidden’ medical curricula influence medical students’ perceptions towards careers in primary care. It defines the ‘formal curricula’ as the structured education that takes place within the classroom setting. By ‘informal curricula’, it refers to learning programmes and activities that occur outside the classroom and that include informal interactions with medical professionals. It refers to the ‘hidden curricula’ as the implicit or unspoken messages that are communicated in the students’ educational and training experience. These influences may occur at an organisational or cultural level [12]. Based on the findings, the article proposes recommendations on how different stakeholders might collaborate in strengthening the medical school experience.

## Methods

### Sample and setting

Purposive sampling was used to ensure that participants came from both the ‘pre-clinical’ and ‘clinical’ years of study allowing us to explore the entirety of the family medicine curriculum. Two focus groups were conducted in each of the three schools. One group consisted of eight to 10 Year 1 or 2 students. The other group comprised eight to 10 Year 3 to 5 students. The students were recruited through student representatives and announcements made by researcher HZ at the end of lectures. Ethical approval was obtained from Nanyang Technological University Institutional Review Board (IRB Reference Number: IRB 2018–07-007).

### Data collection

Participants read a study information sheet before giving written consent. All the focus groups were conducted in English, were led by researcher HZ and facilitated by a co-worker. Each focus group lasted approximately 90 min. The focus groups covered areas related to the students’ career aspirations, their perceptions of careers in primary care, and their clinical postings (Table 2). The themes were organised based on a similar framework used in a report published by National Health Service and Medical Schools Council of England [13]. The discussions were digitally recorded, transcribed verbatim and the scripts were closely reviewed by researchers HZ and HES to ensure accuracy of transcription.

**Table 2:** Focus group questions

Career-related questions:	
What are your career aspirations?	
Have your professional aspirations changed since joining the medical school? If yes, how so?	
To date, how much experience have you had of General Practice/ Family Medicine?	
Career in General Practice/ Family Medicine:	
What opportunities offered by General Practice/ Family Medicine would attract medical students to this field of medicine?	
What are the characteristics of a career in General Practice/ Family Medicine that are challenging?	
Some countries are facing the problem of attracting medical graduates to train in Family Medicine. Why might this be the case? What is your understanding of the situation in Singapore?	
Clinical postings:	
To what extent has your exposure to General Practice/ Family Medicine through your clinical postings confirmed or deterred your interest in this field of medicine?	

### Data analysis

The two researchers read the transcripts independently and adopted a qualitative descriptive approach. This was the preferred method given the aim of achieving straight descriptions of phenomena without “a conceptual or otherwise highly abstract rendering of data” [14]. The straightforward interpretation process also facilitated a relatively easy consensus between the researchers. Using qualitative content analysis, the researchers derived codes inductively from the data and exercised flexibility in the process of analysing and accommodating new insights about the data. The trustworthiness of results was ensured by the collection of data from 3 schools with distinct course organisation and curricula,^4^ by comparing findings with current literature on primary care education and training in Singapore [2, 15], and cross-checking the information provided by the students with key stakeholders. The stakeholders included the Deans and Family Medicine faculty of each school and the staff from MOH Holdings who has been organising interviews for Family Medicine residency selection, as well as soliciting feedback from members of the Study Advisory Board, which comprised the President of College of Family Physicians Singapore, President of Singapore Medical Association and Program Director for the Singapore Health Services (Singhealth) Family Medicine Residency Program.

## Results

Fifty-four students from YLL, LKCMedicine and Duke-NUS responded to the call to participate in the focus groups with 18 students coming from each school. About half of the students (52%) were preclinical and 57.4% were males. Overall, 13 students (24%) expressed some interest in pursuing a career in primary care, but it was not necessarily their top choice of career. The career options attracting greater interest included paediatrics, emergency medicine and obstetrics and gynaecology. The respondents shared eight key concerns and three positive views of pursuing primary care careers based on their medical school experience.

### Concerns arising from formal curriculum

#### Limited professional opportunities

The students expressed concerns about the perceived limited professional opportunities offered by careers in primary care, as captured in the following excerpts:

The amount of funding and emphasis on research and innovation for FM is not as much as the other specialty … Let’s say you are trying to apply for a clinical research grant, if you are an FM physician, there’s almost no way you’re going to get such a grant.

Actually at this stage I don't think there has been much opportunities specifically for family medicine by the government or by the school. For each specialty, there are the SIGs [Special Interest Groups],^5^ there are research opportunities, but not specifically for family medicine.

#### Lifestyle benefits emphasised rather than professional characteristics

Another reason for the lack of popularity of primary care careers appears to arise from a paucity of information about the attractive aspects of the professional or clinical work. The students recounted how careers in primary care were primarily portrayed as having a good work life balance. They recalled being told about positive lifestyle factors but not the professional content and impact on people’s wellbeing.

I feel that FM should be framed, not just always promoting the, oh, there’s great work-life balance, you can go home at 4:00, start work at 8:00, take two-hour lunch break, that kind of thing. Those are just secondary stuff. If you look at it at a broader level, it should be because these are our health needs. And we do not want to burden our expenditure, our health, by having all these complicated procedures.

I feel like people in Medicine, usually, are all quite intellectual people, they’re smart and would like take pride in the work that they have. If the main selling point of Family Medicine is just, it’s got a good lifestyle, I think it would be quite a turnoff for a bunch of people like us, who would like to see ourselves as smart individuals.

#### Lack of training on business acumen

The challenge of managing the business aspects of a private clinic was not a welcome challenge. Students recognised that their training did not prepare them with the relevant skills, and they had concerns about their own abilities to make a business financially viable.

From my past weeks of GP posting experience, I noticed that one thing that always bothers the private GPs is the pharmacy aspect; that they have to manage their own drugs. It is predicated for doctors which drugs to procure and how much they must buy, so it’s not only medicine, you’ve got to think business as well.

I am not inclined to pursue Family Medicine because we are not equipped with the necessary skills to run our own business if we were to go private. There are going to be some hurdles because you are basically a businessman, and you need to be able to sell to your patients why your clinic is better than the other neighbourhood clinics that are within three minutes’ walk.

#### Conflicts created by business in clinical care

Some of the students viewed the profit motivation of private family doctors as undermining the value of primary care and distorting clinical care:

I worked in a private hospital before. I realised that everything in a private hospital operates like a business. There’s a fine line between business and helping the people, so if you go to the GP, you know that they earn most of their money through the medications. They mark up the price quite a lot … Everybody who starts medical school has an interview question about wanting to help others … in the end, what’s more important to you? Is it the patient or the business?

If you are to start your own clinic, it becomes a lot of administrative... more the business handling side rather than anything, so you can be distracted by the business handling side.

### Concerns arising from informal curriculum

#### Mundane case mix

Students repeatedly referred to the cases presenting to family doctors as common minor illnesses. They described these clinical encounters as mundane and for the exceptionally more complex cases, they perceived the primary care role as being limited to onward referrals.

The job might be slightly mundane when you keep giving people MCs (Medical Certificates), or you’re just treating them for a cough, or you see a case that you think is very interesting but you don’t have the resources to treat, so you just send it out to A&E (Accident and Emergency department).

I can’t imagine myself in a GP’s position … If you come across a rare case, you just send it to the hospital, so you always see the same thing and don't really grow as a doctor. It’s as if you’re doing the primary cases and then anything else that’s more advanced goes to the other doctors.

#### Lack of continuity of care

Students also discussed their observations of lack of care continuity during their postings and how this deterred them from pursuing a career in primary care. Their comments point to concerns about the disempowered position of family doctors and their role as solely gatekeepers to care:

A lot of times they would come up with a working diagnosis. Then the next step would just be referral, which is somewhat upsetting to me. I would like to be more involved in the patient’s care and their journey. So, that is something that would pull me back from wanting to join Family Medicine.

In Singapore, we don’t really have such a thing as a doctor taking care of a patient. For GPs, it’s just touch-and-go, they just see you for flu, that’s all. And maybe for another flu, they might go to another doctor. So, no continuity.

#### Limited consultation time

Time pressures and heavy workload formed other concerns, apparent particularly when on placements in public primary healthcare clinics (or ‘polyclinics’).

I think one major challenge is the time for patient interaction because at the polyclinic, the consultation time is just for five minutes, and you feel like the doctor is also being pressured to just keep moving the patients.

Primary care has lost its touch. Polyclinics are just funnelling out patients. I see them probably spending no more than three or four minutes with each patient. You can’t have a meaningful bond with your patients. That’s what primary care is supposed to be. You’re supposed to care for the family.

### Concerns arising from hidden curriculum

#### Specialists’ negative attitudes towards family doctors

Disparaging remarks from specialists that careers in primary care are not as prestigious as specialist careers have largely influenced the students’ career choices. During hospital postings, specialists spoke in condescending terms about the effectiveness and quality of doctors working in primary care, creating a poor and lasting impression on students. The excerpts below highlight this sentiment:

Before I went to my surgery rotation, I was actually quite interested in Family Medicine. Then during a surgery rotation, all the surgeons were telling me, oh, look at what I can do to the patient. If you’re in Family Medicine, you can’t do this. And actually, that has made me not as inclined to Family Medicine because I realise that a lot of problems that the patients present with, I might not be able to do something.

I’m hesitant to pursue Family Medicine because when I’m attached to the specialties; they bitch on the Fam-Med guys for giving referrals they think are substandard … I was in clinic with an O&G doctor and he got a referral for a cervical polyp from the polyclinic. He did the physical exam and was like, that’s not polyp, so stupid. They bitch on how the Fam-Med guys don’t know what they’re doing, or they’ve lost touch or not sufficiently skilled … It seems like they sometimes think of them as incompetent and unable to manage patients appropriately … So, I have a fear that that might happen if I go into Family Medicine.

### Positive perceptions arising from informal curriculum

The students’ perceptions of what careers in primary care have to offer are not entirely negative; they also articulated positive views about primary care, as cited below.

#### Opportunities for entrepreneurialism and a portfolio career

Some students shared that they were attracted by the opportunities for enterprise:

My exposure with Family Medicine is really through my house tutors. So, my house tutor is a GP and he represents quite a few groups because he is part of a larger healthcare group. He also has his own clinic, and now as a house tutor he is also quite clinical with teaching as well. He shared many of his experiences about how his life is as a GP, the pros and cons. That was quite insightful. Previously I would think that as a GP, the community cases you see are quite run off the mill, quite a standard set, so I thought it can be a bit boring.

I realise that family medicine is actually broader than I thought that it was. I thought that it was just that you become a polyclinic doctor or you set up your own clinic, but then because my doctor was doing more corporate things, so he taught me a lot about running his business, how he works with partners and sets up the clinic, how they expanded and set up even more clinics.

#### Breadth of clinical problems presented

Students who had witnessed patients with problems other than common minor self-limiting cases were more receptive to considering a career in primary care. These are the students who had placements at clinics that had a wide variety of patients, both clinically and demographically:

I saw quite a wide variety of cases because the patients in my GP ranged from bankers to construction workers since they are all under insurance schemes. So, it was quite cool. I’m happy to say that only 50% of my patients had ‘flu. The rest had injuries from work, and because they travelled, infectious disease and stuff like that. So that was a lot broader.

I feel that the area where the GP clinic is based in plays a part in what kind of patients you see. For example, in one neighbourhood, most patients just come in to get MCs, but then in Little India, where there are a lot of Indian construction workers, they present with a whole lot of different presentations. It was much more interesting in terms of the cases that the doctor sees and the kind of challenges he faces. For example, not a lot of them are insured, and they struggle to pay the consultation fees, and even if they were supposed to go to A&E or for a follow-up, they didn’t go because they couldn’t pay.

### Positive perceptions arising from hidden curriculum

#### Improved future for primary care

The students perceived that places on the Family Medicine Residency Programme were becoming more desirable and increasingly competitive with the government’s greater emphasis on the need for primary care with an ageing population.

I heard from my friends in other medical schools that residency slots in FM are limited. Things have changed. FM was the last choice but now, it’s becoming a very popular choice.

I think FM is increasingly becoming a competitive specialty. In the future, there might be more opportunities for FM. Because of the ageing population, they’re pushing towards primary physicians.

## Discussion

We found that many of the Singaporean students’ perceptions of careers in Family Medicine were shared with other countries’, particularly the perceived mundane clinical encounters of a family doctor and the low status of a generalist career vis-à-vis that of a specialist one. These have been reported in Australia, Canada and the United Kingdom [8, 9, 11, 16]. While many other works have framed work-life balance as a perk of the primary care profession [9, 11, 16], this study corroborates Parker et al.’s [10] research in New Zealand, which reports that although students find flexible working conditions an attractive part of a primary care career, they would like more emphasis given to the clinical aspects of primary care.

In addition, our study has highlighted the importance of identifying the current limitations of different aspects of the Family Medicine curricula in Singapore’s medical schools. It has underscored issues beyond the formal curriculum that may have significant implications on students’ perceptions of primary care and their career choice. Our findings have also demonstrated that as much as their perceptions are influenced by their medical school experience, the weaknesses in promoting careers in primary care need to be addressed through the concerted effort of various stakeholders. While previous studies have shed light on the initiatives that can be implemented by different stakeholders, they were discussed only briefly. More focus was given on the role of medical schools without explaining how state institutions and professional bodies can contribute to improving the recruitment of future graduates into the primary care workforce [16–20]. Students’ concerns point to the need to tackle the negative and powerful impact of informal and hidden curricula on interest in primary care, as well as broader policy initiatives.

### Recommendations

Findings from the interviews have shown that perceptions of primary care careers being mundane and lacking in continuity of care are widespread among the students. This may be attributed to the lack of exposure to a variety of different primary care facilities and the short duration of their Family Medicine postings. Both preclinical and clinical students commented that their exposure to primary care was too little for them to make any informed career decision about General Practice or Family Medicine.

To expose students to the breadth and complexity of primary care requires attachments in a wider variety of primary care locations during their Family Medicine attachments, including private clinics that attend to a wider socio-demography of patients. Another way is to increase the amount of time spent in primary care placements and to consider the format of that time. Longitudinal placements have been favoured in other countries to enable students to appreciate the importance of continuity of care and the management of long-term conditions [13]. Such placements also expose students to a wider case mix. A study conducted by Koehler and McMenamin on medical students’ perceptions of a career in General Practice conducted in Australia demonstrates that students who had more than 80 h of GP placement experience were more likely to appreciate a diversity of patients’ illnesses [9]. The benefit of such placements is enhanced if GP tutors provide timely feedback to students which enhances their professional development and encourage reflective practice [19].

To heighten students’ awareness and knowledge of what a career in primary care entails, changes are needed to adjust the informal curriculum from negative to positive. Beyond sharing the job scope, career trajectory and lifestyle benefits of a career in primary care, GP teachers need encouragement to also discuss the value of their work to patient’s well-being as their specialist colleagues do. Drawing students to the core aspects of primary care will be more valuable than promoting positive lifestyle perks, which research has deemed less challenging and stimulating to students [10]. Imparting the value of primary care as the foundation of healthcare and championing the vision of the profession as intellectually challenging and rewarding may help students better understand the contribution of primary care, and increase their ability to make informed career decisions without feeling pressured by society or their peers.

Consideration also needs to be given to the influence of role models on the informal curriculum. When sharing their professional and personal experiences with the students, GP teachers need to refrain from framing their career choice as an inevitable outcome of their failure to get into a specialty so as not to reinforce the perception that primary care professions are substandard.

Studies from Australia and Canada have shown that students and young doctors value in their role models integrity, compassion for patients and a positive attitude to junior colleagues [21, 22]. Also identified as positive attributes were clinical competency, enthusiasm for the subject and teaching ability. Those doctors who were rated highly as role models were the ones who spent more time teaching and who emphasised the importance of the doctor-patient relationship. Enhancing the visibility of GPs as teachers and supporting their development as educators and as examiners is a task that would be best tackled locally by the medical schools in collaboration with the College of Family Physicians Singapore (CFPS). Attention could also be given to how the residents in training could become greater role models to the undergraduate population.

Furthermore, measures are needed to tackle the undermining of primary care as a career. Refining mechanisms to enable students to report safely on any disparaging remarks encountered in formal teaching or on clinical attachments is only part of what is required [13]. In parallel, there needs to be initiatives to generate professional respect amongst doctors and faculty, teaching of students the skills needed to be assertive and to challenge denigration, and creating a positive culture of respect for colleagues within healthcare. To achieve this successfully will require collaboration between the medical schools, healthcare providers and professional bodies including the Singapore Medical Council (SMC) and Singapore Medical Association (SMA).

#### Strengths and limitations

This large qualitative study examines the perceptions of students from all three medical schools in Singapore. It contributes to the growing body of international literature from countries such as the United Kingdom, Ireland, New Zealand, Australia, Japan and Malaysia on medical students’ attitudes towards a career in primary care but informs us specifically about issues important in Singapore [8–10, 13, 16, 19, 23, 24]. Each focus group achieved diversity in genders, ethnicities and years of study, which contributed to a rich data. By exploring how different aspects of the medical school curricula influence their perceptions of careers in Family Medicine, it helps to identify gaps and propose recommendations in areas where improvements in primary care education can be made.

The study’s limitation lies in the recruitment strategy. At each school, we entered the field with the aim of purposively recruiting students from different years of study within each focus group. Unfortunately, due to scheduling difficulties, two of the six focus groups only had students from a single year, reducing the interaction and diversity that we were aiming for. As a qualitative study it does not have generalisability and as a cross sectional study, it limits our ability to track change over time. However, the findings from this qualitative study has informed the design of a quantitative survey to medical students, which will enable us to estimate the prevalence of experiences and attitudes, as well as to explore the relationships between socio-demographic factors and perceptions.

A potential line of future research would be to conduct a longitudinal study on how the students’ attitudes towards a career in this field of medicine may have changed after their postings in primary care, and the factors that may have influenced this change. Future research might also usefully incorporate the views of family doctors to evaluate whether or not their career experiences challenge or affirm the students’ concerns about pursuing a career in primary care. Further studies that explore contemporary facilitators and barriers to family doctors’ engagement with undergraduate and postgraduate education should also be conducted.

## Conclusions

Our study has explored the factors that influence the attitudes of medical students towards a career in primary care. While factors such as perceived lack of prestige of this profession have also appeared in other studies, the findings point to the causes of such attitudes. They highlight that the students’ concerns are largely attributed to the lack of exposure to primary care within the formal curriculum, and negative issues within the informal and hidden curricula of medical schools. Despite these concerns, their overall perception of the training programmes in primary care in Singapore is generally positive, which suggests that what needs to be addressed are the gaps in the medical school experience. More broadly, this study highlights the need for a collaborative approach involving tertiary education, professional bodies, healthcare providers and health policy to improve the overall prestige of General Practice and Family Medicine in Singapore. Ultimately, the recruitment and contribution of doctors to the primary care workforce can only be improved with stronger collaboration among various stakeholders.

## Data Availability

The datasets used and analysed during the current study are available from the corresponding author on reasonable request.

## Abbreviations

MOH: Ministry of Health
YLL: Yong Loo Lin School of Medicine
LKCMedicine: Lee Kong Chian School of Medicine
Duke-NUS: Duke-NUS Medical School
GP: General Practice
FM: Family Medicine
CFPS: College of Family Physicians Singapore
SMC: Singapore Medical Council
SMA: Singapore Medical Association

## Footnotes

1 A general practitioner can be any doctor in General Practice whereas a family physician must possess at least a postgraduate qualification such as Graduate Diploma in Family Medicine (GDFM) or Masters of Medicine in Family Medicine (MMed FM) before he or she can be admitted to the Register of Family Physicians [4].

2 The Family Physician Register was formally established in 2011 and listing only began from 1st January 2012 onwards. The latest figure is based on the total number of family physicians to date as reported on the websites of Singapore’s Ministry of Health and Singapore Medical Council.

3 The three-year programme equips its trainees with focused and structured educational and clinical experiences in the hospitals and polyclinics, as well as sessions that combine foundation teaching, continuity clinics, skills workshops, attachments and community medicine [7]. The number of training spots does not necessarily equate to the number of matched applicants. In some years, there may be more training positions than matched applicants because some candidates have applied for more than one residency programme and were matched to a non-Family Medicine programme.

4 Family Medicine began to be taught as a formal discipline in the undergraduate curriculum at YLL in 1987. The academic Family Medicine department in the other two schools was developed much later with the establishment of Duke-NUS in 2005 and LKCMedicine in 2013 [2].

5 SIGs are Special Interest Groups that aim to be a platform through which medical students can gain insights about a particular field of medicine, such as the scope of the specialty and what the discipline entails.

## References

[1] Thomas SL (2013). Family medicine specialty in Singapore. J Family Med Primary Care.

[2] Goh LG, Ong CP (2014). Education and training in family medicine: progress and a proposed national vision for 2030. Singap Med J.

[3] Tan KB, Earn LC (2019). Integration of primary care with hospital services for sustainable universal health coverage in Singapore. Health Systems Reform.

[4] Singapore Medical Council. Family Physician Registration. 2018. https://www.healthprofessionals.gov.sg/smc/becoming-a-registered-doctor/register-of-medical-practitioners/family-physician-registration. Accessed 19 Sept 2020.

[5] Singapore Medical Council. Annu Rep 2018. 2018. https://www.healthprofessionals.gov.sg/docs/librariesprovider2/publications-newsroom/smc-annual-reports/2018-smc-annual-report.pdf. Accessed 19 Sept 2020.

[6] Singapore Medical Council. Family Physician. 2020. https://prs.moh.gov.sg/prs/internet/profSearch/mgetSearchSummaryByName.action. Accessed 19 Sept 2020.

[7] Family Physicians Accreditation Board. Family medicine residency Programme. 2018. https://www.healthprofessionals.gov.sg/fpab/becoming-a-family-physician/family-medicine-residency-programme. Accessed 19 Sept 2020.

[8] Reid K, Alberti H (2018). Medical students’ perceptions of general practice as a career; a phenomenological study using socialisation theory. Education Primary Care.

[9] Koehler N, McMenamin C (2016). Flexible but boring: medical students’ perceptions of a career in general practice. Education Primary Care.

[10] Parker J, Hudson B, Wilkinson T (2014). Influences on final year medical students’ attitudes to general practice as a career. J Primary Health Care.

[11] Scott I, Wright B, Brenneis F, Brett-MacLean P, McCaffrey L (2007). Why would I choose a career in family medicine?: reflections of medical students at 3 universities. Can Fam Physician.

[12] Jeffrey D (2014). Medical mentoring: supporting students, doctors in training and general practitioners.

[13] Wass V, Gregory S, Petty-Saphon K. By choice—not by chance: supporting medical students towards future careers in general practice. London: Health Education England and the Medical Schools Council; 2016. https://www.hee.nhs.uk/sites/default/files/documents/By%20choice%20-%20not%20by%20chance%20PDF.pdf Accessed 19 Sept 2020.

[14] Sandelowski M (2000). Whatever happened to qualitative description?. Research Nurs Health.

[15] Wong TY, Cheong SK, Koh GC, Goh LG (2008). Translating the family medicine vision into educational programmes in Singapore. Ann Acad Med Singap.

[16] Barber S, Brettell R, Perera-Salazar R, Greenhalgh T, Harrington R (2018). UK medical students’ attitudes towards their future careers and general practice: a cross-sectional survey and qualitative analysis of an Oxford cohort. BMC Med Educ.

[17] Barber JRG, Park SE, Jensen K, Marshall H, McDonald P, McKinley RK (2019). Facilitators and barriers to teaching undergraduate medical students in general practice. Med Educ.

[18] Henderson M, Upham S, King D, Dick ML, van Driel M (2018). Medical students, early general practice placements and positive supervisor experiences. Educ Primary Care.

[19] O'Regan A, Culhane A, Dunne C, Griffin M, McGrath D, Meagher D (2013). Integrating postgraduate and undergraduate general practice education: qualitative study. Educ Primary Care.

[20] Sturman N, Régo P, Dick ML (2011). Rewards, costs and challenges: the general practitioner’s experience of teaching medical students. Med Educ.

[21] Wright S (1996). Examining what residents look for in their role models. Acad Med.

[22] Lublin JR (1992). Role modelling: a case study in general practice. Med Educ.

[23] Ng C-J, Leong K-C, Teng C-L (2005). What do medical students think about primary care in Malaysia? A qualitative study. Education Primary Care.

[24] Ie K, Murata A, Tahara M, Komiyama M, Ichikawa S, Takemura YC (2018). What determines medical students’ career preference for general practice residency training?: a multicenter survey in Japan. Asia Pac Fam Med.

