# Correction to: Medical students’ attitudes towards careers in primary care in Singapore

**DOI:** 10.1186/s12909-021-02707-7

**Published:** 2021-05-10

**Authors:** Humairah Zainal, Helen Elizabeth Smith

**Affiliations:** Department of Family Medicine and Primary Care, Lee Kong Chian School of Medicine, Nanyang Technological University, Singapore, Singapore

**Correction to: BMC Medical Education 20, 464 (2020)**

**https://doi.org/10.1186/s12909-020-02377-x**

Following publication of the original article [1], the authors informed us that an incorrect grant number was reported in the **Funding** section. The corrected text should read as follows:

Funding

This study was supported by National Medical Research Council (NMRC) Health Services Research Grant (NMRC/HSRG/0093/2018).
